# Psychologists’ Perspectives of Service-Access Barriers for Syrian Children with Disabilities in a Turkish Refugee Camp

**DOI:** 10.3390/healthcare14193258

**Published:** 2026-10-01

**Authors:** Ibraheem M. Alsawalem, Abdulmalik Alkhunini

**Affiliations:** 1 Special Education Department, College of Education, University of Hail, Hail 55476, Saudi Arabia; 2 Special Education Department, College of Education, Imam Mohammad Ibn Saud Islamic University (IMSIU), Riyadh 11432, Saudi Arabia

**Keywords:** refugee children with disabilities, educational access, physical and mental healthcare access, psychosocial support, trauma and displacement

## Abstract

**Background/Objectives:** Syrian refugee children with disabilities face multiple barriers to accessing essential healthcare, education, psychological support, and protection services. These challenges are often compounded by displacement, poverty, language and documentation difficulties, and limited specialized services. This study aimed to explore these barriers and their interrelated challenges exclusively from the perspectives of practitioners working directly with this population within a refugee-camp context. **Methods:** A qualitative case study design was employed, utilizing purposive sampling to recruit 10 Syrian psychologists working directly with children with disabilities in a refugee camp in Hatay Province, Turkey. Data were gathered through semi-structured interviews and analyzed using Braun and Clarke’s six-step thematic analysis. Data were therefore derived exclusively from practitioners’ perspectives and did not include direct interviews or other data collected from children or caregivers. **Results:** Five interconnected domains of barriers were identified: trauma and mental health; limited access to general and specialized healthcare; documentation, language, and educational-system barriers; poverty and child labor; and unsafe living conditions and inadequate protection. Participants also described substantial family-level economic and caregiving pressures. However, because children and caregivers were not interviewed, the findings represent practitioner-informed perceptions rather than direct accounts of lived experience or population-level prevalence estimates. **Conclusions:** This study contributes to the limited literature by illustrating, from practitioners’ perspectives, how disability, displacement, service-access constraints, and protection risks interact within a refugee-camp context. The findings support the development of inclusive and trauma-informed services, proactive screening and outreach initiatives, family-centered support, and coordinated responses across health, education, protection, and humanitarian sectors.

## 1. Introduction

The protracted Syrian conflict has resulted in the displacement of millions of individuals, including a substantial number of children, to neighboring countries. Turkey has become a major host country for this displaced population, with more than 3.5 million Syrian refugees estimated to be residing there by late 2024; approximately half were children [1]. Geographical proximity, an initial open-door policy, and temporary protection frameworks drove this migration [2]. Consequently, millions settled in urban areas and camps, frequently facing substandard living conditions and severe barriers to essential services, particularly for children with disabilities. While extensive literature documents these broad challenges, a critical research gap persists regarding how service access barriers are experienced and interpreted on the ground. To address this gap, the present study explores the structural barriers to healthcare, education, and psychological access exclusively through the professional perspectives and interpretive lenses of Syrian psychologists working directly within a camp-based setting.

Disability prevalence among this displaced population is notably high, with approximately 28% of individuals aged two and older presenting with a disability [3]. In the cited breakdown, physical impairments accounted for 44.2%, sensory impairments including vision and hearing for 42.4%, and neurodevelopmental, intellectual, or cognitive conditions for 13.4% [4]. Importantly, this published breakdown does not separately identify psychosocial disabilities. Their omission should not be interpreted as evidence that psychosocial disabilities are absent; rather, it reflects a limitation in the available categorization and reinforces the need for disability statistics to include psychosocial conditions explicitly. Because these figures refer to the broader surveyed population and not to the study camp, they should not be read as an estimate of the proportion of children with disabilities within the 14,000 camp residents.

Furthermore, the catastrophic February 2023 earthquakes, affecting over 16 million people, compounded these hardships, obstructed repatriation efforts [1,5], and amplified the vulnerability of disabled refugee children [6,7]. Natural disasters severely impact adult populations, including educators expected to re-establish normalcy within schools despite experiencing personal trauma and displacement [8]. Despite widespread humanitarian interventions, systemic service delivery gaps persist. Turkish government and international NGO initiatives have been implemented, yet systemic barriers continue to impede educational and healthcare access [9]. Consequently, chronic displacement, social exclusion, and psychological distress, including post-traumatic stress disorder (PTSD), exacerbate the complex, long-term vulnerabilities of these children [2,10,11].

## 2. Literature Review

### 2.1. Challenges to Mental and Physical Healthcare Access

Syrian refugee children with disabilities face complex vulnerabilities, experiencing heightened risks of post-traumatic stress disorder (PTSD), anxiety, and depression stemming from war and displacement [9]. Intersecting trauma and disability impede children’s ability to process adverse experiences and adapt to unfamiliar environments [12]. Professional shortages and post-migration stressors further restrict access to adequate psychological care [13]. Extensive research highlights that untreated psychological distress severely impairs social development, academic performance, and community integration [7]. The scarcity of culturally and age-appropriate interventions deepens these developmental hurdles [14,15], while chronic instability and weak support networks amplify long-term cognitive and emotional damage [16]. Conversely, tailored mental health support significantly mitigates trauma and facilitates host-country adjustment [14,15,17].

Simultaneously, structural barriers impede physical healthcare and specialized medical rehabilitation. Turkish healthcare facilities frequently lack necessary equipment [18], while overworked medical infrastructure, linguistic barriers, and cultural divides delay timely treatment [19]. Informal settlements and camps suffer from severe service scarcity [20], and the shortage of Arabic-speaking providers compromises treatment quality [10]. Poverty, undocumented status, and restrictive health insurance policies further restrict access to formal medical systems [21]. Low-income families cannot readily afford assistive devices, surgeries, or specialized therapies, and existing insurance rarely covers comprehensive disability services [18]. Compounded by social stigma and limited legal awareness, these barriers drive profound, long-term developmental consequences and elevate the risk of social isolation [22].

### 2.2. Barriers to Accessing Education

Educational access is another area where Syrian refugee children with disabilities face substantial barriers. While Turkey has made significant strides in integrating these children into the national educational system, they often remain excluded due to a lack of specialized educational services and shortages of qualified special education teachers, the language barrier, and physically inaccessible school facilities [19,23]. Furthermore, the Turkish education system is often unable to meet their needs because they require individualized support and adapted curricula, especially when the children are residing in refugee camps or densely populated urban areas [24].

These obstacles impact children’s social integration and future opportunities through educational exclusion. Research has also demonstrated that the lack of facilities and resources required to deliver quality education in refugee camps make these children especially susceptible to educational gaps [25].

The economic impact of displacement may also have an effect, as children with disabilities from low-income demographics tend to be more likely to encounter significant barriers when trying to access essential services, simply due to the cost associated with such accommodations [26]. Financial constraints often lead to a lack of access to critical services, such as medical treatment, rehabilitation, and special education programs that these children need to thrive, resulting in delayed or inadequate care. Malloy et al. [2] found that in low-income families, interventionists and healthcare professionals are often forced to prioritize basic needs (e.g., food and shelter) over health and educational services, leaving children without complete support. The absence of ongoing financial assistance exacerbates these challenges, leaving families unable to afford consistent care or meet the requirements of their children’s disabilities [27].

### 2.3. Social Well-Being and Physical Safety

The target population also faces widespread social exclusion and marginalization, due to their disabilities and refugee status [8,28]. Research has found that refugee children with disabilities are more likely to encounter discrimination, be isolated from their peers, and have fewer socialization opportunities [23,29,30,31]. This exclusion is especially noticeable in Turkish schools and refugee camps, where negative social perceptions of refugees and people with disabilities can exacerbate existing marginalization.

While explicit stigma is rarely examined as an isolated construct in the literature, studies frequently document related barriers such as bullying, discrimination, and peer marginalization [8,28]. However, studies often fail to clarify how disability-specific stigma operates independently from broader social exclusion in displaced populations.

Furthermore, there are few security requirements in place for the protection of refugee children with disabilities, many of whom reside in refugee camps, making them susceptible to abuse, neglect, and exploitation [2]. These children are also more likely to be exploited under the limited child labor regulations, because families struggling financially may encourage or even require their children to work [11]. The safety, developmental opportunities, and human rights of children with disabilities who experience displacement and financial vulnerability are at risk, necessitating immediate humanitarian and host government interventions [2,11].

### 2.4. Statement of the Problem and Research Rationale

Syrian refugee children with disabilities face numerous structural obstacles that limit their access to healthcare, education, and mental health support in Turkey. This study’s main contribution is empirical and interpretive rather than prevalence-based: it documents how practitioners working inside one refugee camp perceive the interaction of disability-related needs with documentation, language, specialist shortages, poverty, trauma, and protection failures. The study is not a validation study for a data-collection instrument, nor does it claim to measure children’s situation directly. It is a qualitative practitioner-perspective case study intended to identify service-system barriers and generate context-sensitive questions and recommendations for subsequent research with children and caregivers.

While issues related to children’s integration, long-term well-being, and culturally aware interventions remain important gaps in the literature, these topics are beyond the immediate scope of this study. However, these areas represent critical directions for future research to enhance understanding and support for this population. This study focused specifically on identifying the structural barriers encountered by Syrian refugee children with disabilities, through the perceptions of the Syrian psychologists who work with them directly in Turkish refugee camps. Through this focus, we aim to provide practical recommendations to improve service structures and promote more equitable and inclusive approaches.

## 3. Methods

This study was designed and reported in accordance with the Consolidated Criteria for Reporting Qualitative Research (COREQ) 32-item checklist to ensure rigorous and transparent qualitative reporting [32]. Using qualitative interviews, the current study aims to identify cross-sector service barriers as perceived by Syrian psychologists and to clarify how those barriers are thematically related. Semi-structured interviews were used in this qualitative study to gather extensive data about the difficulties faced by Syrian refugee children with disabilities through the insights of Syrian psychologists. According to Creswell [33], interviews are a useful tool for gathering the rich, detailed data needed for interpretive research, as they allow the researcher to obtain insightful information through the viewpoints offered by participants. In addition, semi-structured interviews were deemed appropriate for this study due to their adaptability, which allows for a more thorough examination of the participants’ perceptions and allows the researcher to fully comprehend the lived experiences of those being interviewed [34].

### 3.1. Setting and Participants

The research was conducted in a refugee camp in Hatay Province, near the Syrian–Turkish border. The camp was selected purposively as a critical and information-rich case because it had an established humanitarian service infrastructure, a substantial concentration of children with disabilities, and direct relevance to the post-2023-earthquake humanitarian context. The camp hosted approximately 14,000 refugees, including approximately 255 children identified by the site as having disabilities (about 1.8% of camp residents); this site-level count is descriptive and was not independently verified by the researchers. Eight interviews were conducted in person at the camp and two via Google Meet. The camp was one of approximately 20 refugee settlements across Turkey, so the study was designed for contextual depth rather than national representativeness.

Psychologists were selected as practitioner informants because they had direct professional experience with children with disabilities and could describe service pathways, institutional procedures, and barriers observed across cases. The target eligible population comprised all qualified psychologists working within the camp. Inclusion criteria required participants to be licensed or practicing psychologists actively providing mental health or support services to Syrian refugee children with disabilities within the camp. Exclusion criteria excluded professionals with less than six months of field experience or those not directly engaged with pediatric disability cases.

The participants’ perspectives provide an informed system-level perspective, but they cannot be a substitute for the firsthand accounts of children and caregivers. We initially invited 20 psychologists working with different aid organizations in the camp; 10 consented and participated. Information about the 10 invitees who did not participate—including age, gender, and years of experience—was not collected or was not available to the researchers, so nonresponse bias cannot be assessed. The reasons reported for nonparticipation were operational constraints within aid organizations and the high-stress work environment. Purposive sampling was used to recruit participants who were positioned to address the research topic [35].

These participants included psychologists with experience working with multiple types of disabilities. The sample consisted of two females and eight males, aged 28–46 years, with 5–15 years of experience counseling children with disabilities. All participants held advanced degrees in psychology and had previous clinical experience in Syria before beginning work in Turkey. The sample size was constrained by recruitment and logistical conditions as detailed in Table 1. 

Data saturation was systematically monitored and evaluated by tracking code repetition and thematic stability across the interviews. Saturation was determined to have been reached when no new codes, themes, or conceptual insights emerged from subsequent data collection, indicating a sufficient level of informational redundancy and depth. Specifically, no new insights regarding systemic resource shortages, diagnostic delays, language and documentation barriers, financial constraints, or safety concerns emerged during the final three interviews. Saturation was reached by the tenth interview for these core service-barrier themes, consistent with the context-dependent use of saturation in qualitative interviewing [36]. However, saturation was not claimed for every interview question, every disability category, or the full range of family experiences, acknowledging that the limited sample and single-site design remain important constraints.

The sample size was considered adequate for this focused, purposive qualitative case study because the analytic goal was depth and thematic development within one camp, not statistical estimation. Data saturation was systematically monitored and evaluated by tracking code repetition and thematic stability across the interviews, determining that saturation was achieved when the final three interviews yielded no new codes or novel insights regarding core service barriers. Saturation was reached by the tenth interview for the core service-barrier themes described above, consistent with the context-dependent use of saturation in qualitative interviewing [36]. The study does not claim that 10 interviews exhaust the experiences of all psychologists, children, families, or disability groups in Turkey. Additional camps and participant groups are required to test the transferability of these findings.

### 3.2. Instrument Purpose and Stage of Administration:

The data collection utilized a semi-structured interview guide consisting of 22 open-ended questions developed by the authors (see Supplementary File S1). The primary purpose of this instrument was to explore structural and cross-sector service barriers for Syrian refugee children with disabilities from the perspectives of camp-based psychologists. The interviews were conducted after reviewing relevant literature and study objectives during a field research visit to a refugee camp in Hatay Province, Turkey, following institutional ethical approval [33,34].

### 3.3. Application Method and Administrator:

The first author conducted 10 interviews (8 in person at the camp and 2 via Google Meet), each lasting 30–35 min. Interview questions were shared with participants in advance. When audio recording was permitted and feasible, sessions were recorded and transcribed; otherwise, detailed contemporaneous field notes were taken and expanded immediately afterward. To mitigate potential variations in data quality between audio-recorded sessions and contemporaneous field notes, all handwritten notes were systematically expanded immediately following each interview.

To ensure the credibility and trustworthiness of the qualitative findings, member checking was thoroughly performed. A subset of participants was provided with Arabic descriptions, summaries of preliminary themes, and tentative interpretations derived from their interviews. This process allowed participants to verify accuracy, clarify misunderstandings, and provide feedback, ensuring that the documented findings faithfully reflected their lived experiences, professional insights, and perspectives before final data analysis [37].

### 3.4. Data Collection

The first author had previously participated in a humanitarian support mission in Turkey. This professional relationship provided access to a pool of psychologists with experience working with Syrian refugee children with disabilities and was used only to facilitate recruitment. Twenty individuals were invited; 10 consented to participate. Eight interviews were conducted at the camp during the first author’s research visit, while two participants who were unavailable in person were interviewed online using Google Meet. Recruitment and interview scheduling were based on participant availability and preference. The logistical limitations of a single visit, organizational workload, and the sensitive work environment restricted recruitment and prevented expansion to additional camps during the study period.

### 3.5. Data Analysis

Qualitative data from transcripts and expanded field notes were analyzed using Braun and Clarke’s six-step thematic analysis framework: familiarization, generation of initial codes, searching for candidate themes, reviewing themes, defining and naming themes, and producing the final report. Manual inductive coding was conducted by the first author and managed using NVivo 12 software, while the second author independently reviewed the coded framework and thematic structure to ensure analytical rigor [38].

### 3.6. Researcher Reflexivity

The first author’s prior involvement in humanitarian work in Turkey facilitated access to participants but may also have influenced the interview process and interpretation of participants’ accounts. To enhance reflexivity and reduce potential researcher influence, the interview process remained guided by the semi-structured interview guide, and the second author independently reviewed the coding framework and thematic structure. Member checking was also used to support the credibility of participants’ accounts and interpretations [39].

### 3.7. Ethical Approval and Research Integrity

Given the diverse professional affiliations of the participants, who were all psychologists volunteering with various international humanitarian organizations, including the International Committee of the Red Cross and the Turkish Red Crescent (Kızılay), obtaining unified ethical approval presented significant challenges. To address this, each organization was contacted and asked to provide their ethical review procedures. However, procedural complexities were encountered because the participants operated under different organizational authorities with varying ethical review processes. To address these challenges, established guidelines from international humanitarian research standards were followed. In addition, formal ethical approval was obtained from the Ethical Review Board at Hial University (Approval No. H-2024-131). Furthermore, the researchers strictly adhered to international ethical standards, including the Declaration of Helsinki [40] and the Council for International Organizations of Medical Sciences (CIOMS) Guidelines [41], ensuring that all aspects of the research maintained respect for participants’ rights, good will, and justice throughout the research process. Appropriate consideration was also given to the guidance of the World Health Organization [42].

Culturally sensitive communications were ensured throughout the study, with consideration of the vulnerable status of the refugee children the participants work with and with respect for the cultural norms of the participants. All those interviewed were thoroughly briefed about the study’s purpose, methodology, and potential risks. Informed consent was obtained in writing before each interview. Participation was entirely voluntary; participants were informed that they could withdraw at any time with no consequences or justification. Interview questions were shared in advance. To protect confidentiality and anonymity, all responses were de-identified, and data were securely stored using password-protected digital systems. These measures were essential given the professional vulnerability of the participants and the sensitive nature of the populations they serve. The researchers remained committed to upholding ethical integrity throughout the study and took deliberate steps to ensure participant safety and data protection.

## 4. Results

**Figure 1:**
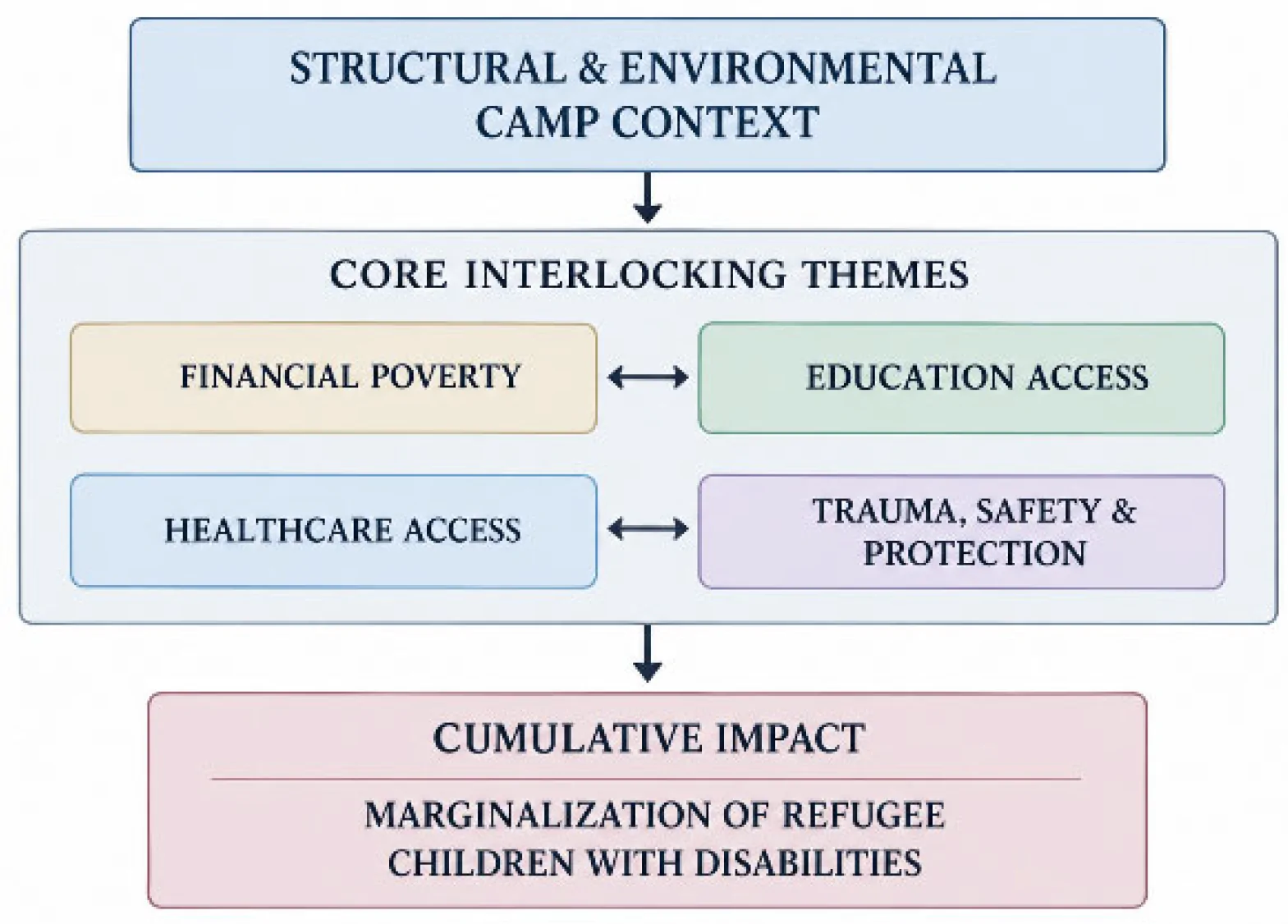
Conceptual map of interconnected service-access barriers reported by Syrian psychologists. Note. This conceptual map illustrates the cross-cutting, mutually reinforcing relationships between the five primary themes as synthesized from the participating psychologists’ qualitative reports.

The primary aim of this research was to identify service barriers as perceived and reported by 10 Syrian psychologists working with children with disabilities in one refugee camp. As such, the results exclusively reflect practitioner observations, clinical interpretations, and professional perspectives; they are not direct reports from children or caregivers, nor do they estimate the statistical prevalence of barriers across the camp or Turkey. Five main themes emerged from the psychologists’ perspectives: trauma and mental health, lack of healthcare access, education access, financial and family pressures, and safety and social protection as detailed in Table 2. Figure 1 presents a conceptual map illustrating the interconnected and mutually reinforcing relationships among these five themes. As described by the participants, the thematic relationships were cross-cutting: poverty and documentation problems restricted education and healthcare; disability-related support needs intensified the effects of language and infrastructure barriers; and unsafe conditions and trauma compounded difficulties in learning and service engagement. Stigma was not coded as a separate theme, but discrimination, bullying, abuse, and marginalization appeared within the education and safety accounts according to the practitioners’ narratives. Participants discussed children with multiple disability types, but the study did not systematically compare single and multiple disabilities; accordingly, no subgroup conclusions are drawn.

### 4.1. Trauma and Mental Health

Drawing on clinical experiences, the participating psychologists consistently reported that the children they work with experience intense and recurring feelings of shock, fear, and anxiety, particularly in response to traumatic events such as the Gaziantep earthquake. According to the practitioners’ perspectives, these emotional challenges significantly affect the children’s psychological wellbeing and everyday functioning. For example, Psychologist 10 described how children often react with immediate fear, sharing, “Anytime a child hears a strange voice, he asks, ‘Are we going to die?’” This illustrates the deep-seated anxiety triggered by their environment, as observed by the clinician. Psychologist 5 also confirmed the prevalence of shock symptoms among their patients, noting that these reactions are widely shared across cases.

Fear was described as a profound issue, manifesting notably through sleep disturbances and nightmares. Psychologist 2 shared the case of a girl who “suffers from fear and dread, especially when sleeping,” experiencing nightmares that vividly recall the traumatic events of war. The interviewed clinicians emphasized that such emotional difficulties are not isolated but appear frequently among these children.

The difficult living conditions in refugee camps—characterized by chaos and scarce resources—were noted by the participants to exacerbate these mental health struggles. Anxiety symptoms are often presented physically, as described by Psychologist 6, who noted a child suffering stomach pains and insomnia related to traumatic memories. Furthermore, the psychologists highlighted that many children find it challenging to express their fears verbally, which can lead to depressive symptoms, including social withdrawal and sadness, as observed by Psychologist 8. The collective perspectives of the participants emphasize the urgent need for mental health interventions tailored to address these intertwined psychological and physical symptoms, recognizing the compounded effects of trauma and environmental stressors on children’s wellbeing from a practitioner standpoint.

The participants’ interpretations emphasize the urgent need for mental health interventions tailored to address these intertwined psychological and physical symptoms, recognizing the compounded effects of trauma and environmental stressors on children’s wellbeing.

### 4.2. Low Supply of Health Services

Analysis of the psychologists’ interviews revealed that, from their professional perspective, refugee children with disabilities encounter barriers to accessing general healthcare, including long wait times. The participants indicated that this issue was likely related to the reality that refugees are simply not a priority within the healthcare system. Psychologist 1 noted, “There are not enough clinics, and Turkish citizens are prioritized over refugees for access to healthcare.” Furthermore, the practitioners stated that limited specialized care and a lack of disability specialists—such as physical and occupational therapists create severe challenges in their daily practice.

Language barriers also affect healthcare access, as observed by multiple practitioners. Psychologist 4 mentioned that Syrian parents of children with disabilities cannot easily access or even make appointments with medical personnel in Turkey because typically there is no Arabic translator available to facilitate this. As reported by the clinicians, without health services provided by Arabic relief agencies in Turkey, refugees are forced to wait a long time to obtain necessary healthcare.

Despite the literature emphasizing the need for mental health support, the psychologists stated that typically these children are not eligible for services. Psychologist 1 stated, “After the earthquake of 2023, the children were traumatized. Our children are suffering but no one helps them.” Other participants described how difficult it is to observe children with disabilities suffering psychologically without any intervention, asserting the need for more psychologists with the skills to work with these Syrian refugee children with disabilities (Psychologist 8). Overall, these practitioner-reported findings reveal systemic gaps that significantly hinder refugee children’s access to both general and specialized healthcare, further exacerbating their vulnerabilities.

### 4.3. Difficult Access to Education

Another theme that emerged from the practitioner interviews was that of educational access. The participants noted that, based on their age, the children they see should be qualified to attend school; however, many of them are not enrolled. Psychologist 3 commented on this issue, saying, “It is hard to register them because they lack identification documents and [typically] do not have a permanent address. Following the registration procedures may sometimes take a year or more.” The interviewed psychologists observed that such delays greatly impact the development of these children.

The language barrier was again cited by the participants as an obstacle to educational access. As Psychologist 4 stated, “Even though some children have been studying in a Turkish school for several years, they still [do not understand] the Turkish language.” This issue was also discussed by Psychologist 9, who said, “Turkish teachers are not interested in the Syrian children, whether [the children] understand the Turkish language or not.” All the participants reported that schools are focused on serving Turkish children rather than accommodating Syrian refugee children. Moreover, the practitioners indicated that this discrimination is perceived to be more serious in the case of Syrian refugee children with disabilities, because not only is there the language barrier, but schools do not provide accessible learning environments to accommodate special educational needs.

The psychologists strongly emphasized the lack of special education programs for these Syrian children with disabilities, noting that they are not included in either inclusive or special education classrooms. Psychologist 7 described the educational environments provided for Syrian refugee children with disabilities as similar to daycare, stating, “There is no actual education for them.” Participants also lamented the lack of identification programs to determine whether accommodations are needed, stating, “No assessments are done to check for disorders associated with the major disability [categories]” (Psychologist 4). As a result, the participants perceived that children with disabilities are left behind, with no access to appropriate education, systematically limiting their opportunities for growth and integration.

### 4.4. Financial Barriers

The financial issues experienced by refugee families also emerged as an important theme from the psychologists’ perspectives. One participant noted, “My patient washes cars and bicycles during the day [just to be able] to meet his basic nutritional needs” (Psychologist 5). This issue was, according to the practitioner, related to the limited financial resources of the child’s parents, which impacts educational progress and development through inconsistent school attendance.

The participants described this challenge using the term “poverty”. One participant said, “To address the complications of disability and achieve an appropriate level of stability, challenges of poverty and financial issues must be removed” (Psychologist 8). The psychologists noted that since fleeing to Turkey, these families have lived in extreme financial hardship, lacking resources to support family members with disabilities. Due to these financial challenges, parents cannot provide essential treatment for their children, as emphasized by a participant who stated, “Parents are not capable of paying for their children’s medicine; the cost is high, and they do not have insurance” (Psychologist 1).

The interviewed clinicians frequently mentioned that the children are “left behind,” reflecting the practitioners’ perception that these refugee families have been largely forgotten. This sense of abandonment is further compounded by financial barriers, intensifying existing challenges and leaving these children and their families marginalized and vulnerable from the perspective of the professionals.

### 4.5. Lack of Protection

The participants also reported a recognized lack of safety in the refugee camps, which, in the case of Syrian refugee children with disabilities, is demonstrated by the violence, abuse, and bullying they experience from both Turkish and Syrian individuals according to practitioner observations. As one respondent shared, “Because of their disabilities, they have been abused and bullied many times inside and outside the camp” (Psychologist 7). The practitioners cited the lack of protection for children with disabilities, noting that life in the camps is “random and rudimentary” and involves no enforced laws or rights to protect refugees. Unfortunately, according to the participants, refugee children with disabilities are considered the weakest and therefore most vulnerable in this setting.

The majority of participating psychologists stated that the living conditions within the refugee camps are insecure and dangerous based on accounts shared with them by their patients. Several also noted that their patients have stated that they feel unsafe inside the camp. To illustrate, one interviewee said, “There is no kind of protection inside the camp, such as [would be provided by] local police or the Syrians who are responsible for managing the camp” (Psychologist 10). The participants expressed deep concern about these conditions, describing them as overcrowded, dirty, inhumane, and “inaccessible for people with disabilities” (Psychologists 3, 5, and 7). These practitioner-reported findings underscore the perceived urgent need for enhanced safety protocols and targeted protection measures to safeguard the rights and wellbeing of Syrian refugee children with disabilities living in camps.

## 5. Discussion

Insights from participating Syrian psychologists reveal severe structural barriers impeding basic service access for Syrian refugee children with disabilities in one Turkish refugee camp. These findings should be framed strictly as practitioner-informed perceptions of service-system functionality rather than direct clinical assessments of children. From their professional standpoint as frontline mental health providers, these psychologists operate as critical bridges—and occasional buffers—between marginalized families and rigid institutional structures. The study’s unique contribution is to show how these barriers cluster across service sectors rather than operating independently: documentation and language barriers affect both school enrollment and healthcare navigation; poverty links treatment gaps with child labor and disrupted attendance; and trauma, unsafe infrastructure, and limited protection reinforce one another. Alternatively, some discrepancies in service uptake may stem not solely from institutional discrimination or policy deficits but from capacity constraints within local agencies, underfunded humanitarian streams, and the complex administrative friction inherent in cross-border displacement management. These findings should be interpreted as a practitioner-informed account of service-system functioning, not as a direct measure of children’s lived experience or the prevalence of psychological symptoms.

The trauma findings parallel those of Aydos et al. [8], whose preschool educator interviews identified infrastructure damage, substandard housing, and absent psychological support post-earthquake. Recent studies have consistently emphasized elevated post-traumatic stress disorder (PTSD) and anxiety rates among displaced children facing compounding natural and man-made disasters [7,15], underscoring the demand for targeted policy interventions.

Participants emphasized that documentation gaps and permanent address requirements obstruct school enrollment. Language barriers further impede academic and medical access, particularly for children with disabilities struggling to acquire Turkish language skills, corroborating Bayraklı [19]. Furthermore, enrolled children encounter societal bigotry and discrimination, echoing findings by Parrott et al. [14], Malloy et al. [2], and Al-Hendawi and Alodat [28] regarding educational exclusion and alienation.

Regarding educational placement, children remain excluded from general and special classrooms due to systemic deficiencies within camp schools [19,24]. Within their professional capacity, the psychologists highlighted an absence of diagnostic identification tools, mirroring wider calls for specialized curricula, enhanced professional roles for school-based clinicians and special educators, teacher training, and improved camp school environments to foster equity and behavioral stability [25,26,29,31].

Healthcare access remains severely constrained by long wait times, linguistic hurdles, and perceived prioritization of Turkish citizens. Camp locations inherently limit medical proximity [20], while local facilities lack adequate specialized infrastructure for pediatric disabilities [18]. The scarcity of Arabic medical translators forces families toward limited relief agency resources [10,19], validating research on how cultural and linguistic divides diminish treatment efficacy [4,13].

The psychologists reported systemic ineligibility for public mental health services, leaving post-earthquake PTSD untreated [16]. Within their professional capacity, these clinicians often attempt to bridge treatment gaps informally, though severe resource scarcity limits their efficacy. Professional shortages, cultural divides, and linguistic barriers hinder effective psychological treatment [13], matching international research advocating for culturally sensitive interventions [14,17]. Paralleling challenges observed in Lebanon [30], economic pressures force children into child labor at the expense of education and health [16].

Uninsured families face critical gaps, as they are unable to afford surgeries, therapy, or assistive devices [18,21], emphasizing the necessity of robust financial aid [2,11,27]. Moreover, participants reported pervasive bullying from both host and refugee communities, reinforcing evidence of social isolation in camps and schools [23]. Overcrowded, unsanitary camp conditions heighten vulnerability to exploitation, abuse, and child labor, demanding urgent humanitarian and governmental protective interventions [2,7,11,15].

### 5.1. Conclusions

This study reveals the multifaceted challenges confronting disabled Syrian refugee children in Turkey based on practitioner reports from a single camp, highlighting trauma, displacement, and systemic barriers. Obstacles such as diagnostic gaps, linguistic hurdles, and economic hardship restrict access to education, healthcare, and psychological support as perceived by the participating psychologists. Discrimination and social exclusion perpetuate a cycle of neglect, severely limiting opportunities for advancement and underscoring the need for tailored intervention frameworks that reflect these localized, practitioner-informed insights.

### 5.2. Implications

The findings present critical implications for policymakers, humanitarian agencies, and educators. Legal mandates should explicitly require inclusive special education and specialized resources. Policy should also prioritize accessible medical infrastructure, qualified interpreters, and specialized care pathways. Integrating psychological services, particularly for post-traumatic stress disorder, into standard humanitarian response plans is essential to mitigating long-term trauma. Services should be designed with a family lens, including caregiver information, referral support, financial assistance, and safeguarding mechanisms that reduce the burden on parents and siblings. Because the study did not directly assess families, these recommendations should be co-designed and evaluated with children and caregivers in future work.

Furthermore, proactive screening, mobile diagnostic initiatives, school-based outreach, and community health-worker referral mechanisms are vital for reaching unregistered, geographically isolated, or otherwise marginalized children who are not receiving services. Strengthening social-protection frameworks, including direct financial aid and measures to reduce child labor, may alleviate economic pressures. Combining proactive outreach with legislative advocacy and accessible complaint and protection mechanisms can help connect children with disabilities to essential support structures. The present findings support these priorities but do not establish their effectiveness; implementation research is needed.

Given that this study was conducted within a single refugee camp, the transferability of these implications is inherently limited; unique local administrative frameworks, camp-specific infrastructure, and localized environmental stressors may mean these strategies require careful adaptation before being applied to other camps, informal settlements, or urban refugee contexts across Turkey.

### 5.3. Limitations

This study possesses several limitations that warrant consideration when interpreting the findings. First, data were collected only from 10 psychologists and not from children or caregivers. Practitioner perspectives offer valuable system-level insight but cannot fully capture children’s lived experiences, agency, family impacts, or the perspectives of parents with psychosocial disabilities.

Second, only 10 of the 20 invited psychologists participated. This 50% non-response rate introduces a potential source of self-selection and non-response bias. Practitioners who agreed to participate may have possessed stronger institutional backing, differing workloads, or heightened engagement with systemic barriers compared with non-participants. Because data regarding non-participants’ demographic and professional profiles were unavailable, direct comparative assessment was not possible. Non-participation was primarily driven by acute operational pressures, high-stress clinical workloads, and administrative constraints within aid organizations rather than systematic professional differences. Consequently, the findings reflect the informed perspectives of accessible practitioner-informants rather than a statistically representative consensus of all camp personnel.

Third, saturation was reached only for the core barrier themes, and not necessarily for every interview question, disability category, or family experience; a larger and more diverse sample may have identified additional themes. A further limitation is that the interview guide placed greater emphasis on barriers and challenges than on facilitators or positive experiences. This focus may have both shaped the issues participants discussed and contributed to the prominence of barrier-related themes in the findings. Therefore, these themes should be interpreted within the context of the interview guide and viewed as reflecting participants’ reported experiences and perceptions.

Fourth, the research was restricted to a single refugee camp in Hatay Province near the Syrian–Turkish border. While chosen for its established humanitarian infrastructure, high concentration of vulnerable populations, and relevance to post-2023-earthquake humanitarian conditions, it remains a localized case study. The service arrangements, the environmental stressors, and the prevalence and severity of post-traumatic stress may reflect specific geographic conditions of this location, which may differ from other camps and urban settlements across Turkey. While this singular focus provided the depth required for a qualitative exploration of systemic service gaps, it limits the transferability of the findings to more stable or geographically distinct settings.

Fifth, the analysis did not systematically compare children with single versus multiple disabilities, and multiple disabilities may present distinct access barriers.

Sixth, some interviews could not be audio-recorded, requiring reliance on contemporaneous and expanded field notes. Although member checking and investigator review were used, this may have reduced the completeness and detail of the data captured.

Finally, the study reports qualitative themes and participant perceptions rather than population estimates, causal effects, or independently verified clinical diagnoses. A family lens is also essential, as parents and siblings may experience increased caregiving demands, financial strain, psychological distress, and difficulty navigating fragmented services. The present study did not directly assess parents’ psychosocial disabilities, family wellbeing, or sibling experiences; these issues are therefore treated as contextual considerations and priorities for future research rather than as findings attributable to the present sample.

### 5.4. Recommendations for Future Research

Future research should prioritize multi-stakeholder approaches by directly engaging refugee children and their caregivers, as their voices are essential for validating systemic findings with lived experiences. To address the nuances of the disability spectrum, subsequent studies must adopt more granular inquiry by stratifying participants according to specific disability categories—such as sensory, intellectual, or physical impairment—rather than treating disability as a monolithic variable. Such research should utilize culturally sensitive, child-friendly methodologies to ensure the inclusion of participants with diverse communication needs, thereby bridging the current gap between practitioner insights and the children’s actual realities.

Furthermore, there is a critical need for large-scale, comparative, and longitudinal research that extends beyond single-site case studies. Future investigations should conduct cross-site analyses across multiple refugee settlements to evaluate how varying local policies and camp management frameworks influence service equity. Longitudinal designs are particularly essential to track the long-term impact of trauma, displacement, and environmental stressors, such as those following the 2023 earthquake, on children’s developmental trajectories. Through the examination of the intersectionality of disability, socioeconomic status, and refugee identity over time, these studies will provide the evidence-based foundation required to develop durable, sustainable, and inclusive support systems.

## Figures and Tables

**Table 1:** Demographic characteristics of study participants.

Pseudonym	Gender	Age	Years of Experience	Interview Format
Psychologist 1	Male	35	10	In-person
Psychologist 2	Female	28	5	In-person
Psychologist 3	Male	42	12	In-person
Psychologist 4	Male	39	11	In-person
Psychologist 5	Male	46	15	In-person
Psychologist 6	Male	30	7	In-person
Psychologist 7	Female	32	8	In-person
Psychologist 8	Male	36	9	In-person
Psychologist 9	Male	29	6	Online (Google Meet)
Psychologist 10	Male	34	8	Online (Google Meet)

Note. “Years of experience” refers to the individual’s years of experience counseling children with disabilities. *N* = 10.

**Table 2:** Summary of themes and quotations from Syrian psychologists.

Main Theme	Sub-Theme(s)	Representative Quotes from Syrian Psychologists
1. Trauma and mental health	(a) Shock and fear (b) Anxiety and depression	Psychologist 10: “Anytime [a] child hears a strange voice, he asks, ‘Are we going to die?’” Psychologist 6: “One child suffers from anxiety, which manifests through stomach pain and difficulty sleeping.” Psychologist 2: “she suffers from fear and dread, especially when sleeping.”
2. Low supply of Healthcare Services	(a) Limited general healthcare access (b) Shortage of specialists (c) Language barrier	Psychologist 1: “There are not enough clinics, and Turkish citizens are prioritized over refugees.” Psychologist 4: “Syrian parents cannot access medical personnel due to lack of Arabic translators.” Psychologist 8: “more psychologists with the skills to work with these Syrian refugee children with disabilities are urgently needed.”
3. Difficulty accessing education	(a) Difficulty with school registration (b) Language barrier (c) Lack of special education services	Psychologist 3: “It is hard to register them because they lack identification documents.” Psychologist 7: “Schools focus on Turkish children; no special education programs for Syrian children with disabilities.” Psychologist 9: “Turkish teachers are not interested in the Syrian children... Children with disabilities are left behind.”
4. Financial barriers	(a) Poverty affecting education (b) Inability to cover medical costs	Psychologist 5: “My patient washes cars just to meet basic nutritional needs.” Psychologist 1: “Parents cannot pay for their children’s medicine; they don’t have insurance.” Psychologist 8: “...challenges of poverty and financial issues must be removed.”
5. Lack of protection	(a) Exposure to violence and bullying (b) Lack of protection in camps	Psychologist 7: “Because of their disabilities, they have been abused and bullied many times.” Psychologist 10: “There is no kind of protection inside the camp, such as local police.” Psychologists 3 and 5: Described camp conditions as “overcrowded, dirty, inhumane, and inaccessible.”

## Data Availability

The study participants did not provide written consent for their data to be shared publicly. Therefore, due to the sensitive nature of the research, supporting data is unavailable.

## Supplementary Materials

The following supporting information can be downloaded at: https://www.mdpi.com/article/10.3390/healthcare14193258/s1, File S1: Semi-Structured Interview Guide.
