# Supplementary material for: Psychologists’ Perspectives of Service-Access Barriers for Syrian Children with Disabilities in a Turkish Refugee Camp
Source: Healthcare (Basel). 2026 Oct 1;14(19):3258. doi: 10.3390/healthcare14193258 (PMC13664849; doi:10.3390/healthcare14193258)
Supplement: Supplementary file 1 [file healthcare-14-03258-s001.zip › healthcare-4535812-supplementary.pdf]

Semi-Structured Interview Guide (22 Questions)

Section A: Professional Background and Context

Can you briefly describe your professional background, qualifications, and years of experience counseling children with disabilities?

1. What are your main roles and responsibilities in working with Syrian refugee children in this camp?
2. What types of disabilities do you most frequently encounter among the children you serve in the camp?

Section B: Trauma and Mental Health

4. How do the traumatic experiences of war, displacement, and disasters (such as the 2023 earthquake) manifest in the children's mental health?

5. What are the most common psychological symptoms (e.g., anxiety, fear, shock, sleep disturbances) observed among these children?

6. How do children with disabilities express or communicate their emotional distress and fears in camp environments?

7. What specialized psychological support services are currently available for these children, and what are their key limitations?

Section C: Access to Healthcare Services

8. What primary obstacles do refugee children with disabilities face when attempting to access general healthcare facilities?

9. To what extent are specialized rehabilitation services (e.g., physical therapy, occupational therapy) available within or near the camp?

10. How do language barriers and the lack of Arabic interpreters affect the delivery of medical and health services?

11. How do health insurance policies, eligibility criteria, and administrative wait times impact timely treatment for refugee children?

Section D: Educational Access and Special Education

12. What administrative or documentation challenges (e.g., lack of IDs, permanent addresses) impede school registration for these children?

13. How does the language barrier affect children's learning outcomes and integration within local educational settings?

14. What special education programs, adapted curricula, or accommodations exist in camp schools to support disabled children?

15. Are formal diagnostic assessments conducted to identify specific learning needs or associated developmental disorders?

Section E: Financial and Family Pressures

16. How does severe household poverty impact the family's ability to secure basic medical treatments, assistive devices, or medication?

17. In what ways do financial constraints force families to engage children in child labor at the expense of schooling or rehabilitation?

18. How do caregiving demands and economic hardships affect overall family stability and support structures?

Section F: Safety, Social Protection, and Recommendations

19. What security and physical protection risks (e.g., violence, abuse, lack of policing) do disabled children experience inside the camp?

20. How do societal stigma, discrimination, and peer bullying impact the social integration and safety of these children?

21. What structural and policy changes are most urgently needed to improve healthcare, educational, and protective access for this population?

22. What key recommendations would you give to humanitarian organizations and host government authorities to build inclusive, trauma-informed services?
